# Progress towards implementation of ACT malaria case-management in public health facilities in the Republic of Sudan: a cluster-sample survey

**DOI:** 10.1186/1471-2458-12-11

**Published:** 2012-01-06

**Authors:** Tarig M Abdelgader, Abdalla M Ibrahim, Khalid A Elmardi, Sophie Githinji, Dejan Zurovac, Robert W Snow, Abdisalan M Noor

**Affiliations:** 1 National Malaria Control, Federal Ministry of Health, Sudan, Republic of Sudan; 2 Malaria Public Health & Epidemiology Group, Centre for Geographic Medicine Research - Coast, Kenya Medical Research Institute/Wellcome Trust Research Programme, P.O. Box 43640, 00100 GPO Nairobi, Kenya; 3 Centre for Tropical Medicine, Nuffield Department of Clinical Medicine, University of Oxford, CCVTM, Oxford OX3 7LJ, UK; 4 Center for International Health and Development, Boston University School of Public Health, 85 East Concord Street, Boston, MA 02118, USA

## Abstract

**Background:**

Effective malaria case-management based on artemisinin-based combination therapy (ACT) and parasitological diagnosis is a major pillar within the 2007-2012 National Malaria Strategic Plan in the Sudan. Three years after the launch of the strategy a health facility survey was undertaken to evaluate case-management practices and readiness of the health facilities and health workers to implement a new malaria case-management strategy.

**Methods:**

A cross-sectional, cluster sample survey was undertaken at public health facilities in 15 states of Sudan. Data were collected using quality-of-care assessment methods. The main outcomes were the proportions of facilities with ACTs and malaria diagnostics; proportions of health workers exposed to malaria related health systems support activities; and composite and individual indicators of case-management practices for febrile outpatients stratified by age, availability of ACTs and diagnostics, use of malaria diagnostics, and test result.

**Results:**

We evaluated 244 facilities, 294 health workers and 1,643 consultations for febrile outpatients (425 < 5 years and 1,218 ≥ 5 years). Health facility and health worker readiness was variable: chloroquine was available at only 5% of facilities, 73% stocked recommended artesunate and sulfadoxine/pyrimethamine (AS+SP), 51% had the capacity to perform parasitological diagnosis, 53% of health workers had received in-service training on ACTs, 24% were trained in the use of malaria Rapid Diagnostic Tests, and 19% had received a supervisory visit including malaria case-management. At all health facilities 46% of febrile patients were parasitologically tested and 35% of patients were both, tested and treated according to test result. At facilities where AS+SP and malaria diagnostics were available 66% of febrile patients were tested and 51% were both, tested and treated according to test result. Among test positive patients 64% were treated with AS+SP but 24% were treated with artemether monotherapy. Among test negative patients only 17% of patients were treated for malaria. The majority of ACT dispensing and counseling practices were suboptimal.

**Conclusions:**

Five years following change of the policy from chloroquine to ACTs and 3 years before the end of the new malaria strategic plan chloroquine was successfully phased out from public facilities in Sudan, however, an important gap remained in the availability of ACTs, diagnostic capacities and coverage with malaria case-management activities. The national scale-up of diagnostics, using the findings of this survey as well as future qualitative research, should present an opportunity not only to expand existing testing capacities but also to implement effective support interventions to bridge the health systems gaps and support corrective case-management measures, including the discontinuation of artemether monotherapy treatment.

## Background

Effective case-management based on parasitological diagnosis and artemisinin-based combination therapy (ACT) is one of the key strategies for the reduction of the *Plasmodium falciparum *malaria burden across the African continent [1]. By 2009, all 42 African malaria endemic countries had changed their policies to support ACT use for uncomplicated malaria. Furthermore, 20 countries had adopted policies promoting all-age group parasitological diagnosis using malaria microscopy and rapid diagnostic tests (RDTs) [2]. Policies in other African countries are under the revision to support parasitological diagnosis.

However, the implementation of effective case-management based on malaria diagnostics and ACTs may face a number of challenges, of which availability of commodities at health facilities and sub-optimal case-management practices are of particular concern. At facilities across Africa it has been shown that ACTs and malaria diagnostics are frequently out of stock [3,4], where diagnostics exist, febrile patients are rarely tested [5-9] and if tested, negative results still result in the prescription of anti-malarials [5,6,9-14]. Furthermore, the use of non-recommended antimalarials is often reported [8,15-18] and the performance of patients' counseling and drug dispensing tasks is rarely optimal [15,17-19]. Failure to ensure the delivery of basic commodities and minimum standards of case-management based on testing and adherence to test results severely compromises the cost-benefit of new malaria case-management strategies [20].

In 2004 Sudan was among the first countries in Africa to change first and second line treatment policy for uncomplicated malaria from ineffective monotherapies to ACTs [21-23]. The first line policy was changed from chloroquine to a combination of artesunate and sulfadoxine/pyrimethamine (AS+SP) and the second line policy was changed from SP to artemether-lumefantrine (AL). For severe malaria parenteral quinine remained the treatment of choice and intramuscular artemether was recommended as second line therapy [24]. Following change of the policy, SP monotherapy was only reserved for intermittent preventive treatment in pregnant women in selected high risk areas.

In 2006, the Sudanese National Malaria Control Programme (NMCP) launched the new 2007-2012 National Malaria Strategy which reconfirmed ACT treatment policy and provided strategic directions to ensure universal access to ACTs and malaria confirmatory diagnosis primarily through microscopy, or RDTs wherever malaria microscopy is not possible [25]. Between 2005 and 2009 the Federal NMCP supported the implementation of the new treatment policy across the 15 states of Sudan. The key implementation activities of relevance for ACT based case-management at the facility level included procurement and distribution of co-packaged AS+SP in paediatric (50 mg AS+500/25 mg SP) and adult (100 mg AS+500/25 SP) formulations, procurement and distribution of co-formulated AL in four weight-specific packages, development and dissemination of treatment protocols and job aids for health workers, orientation sessions and in-service case-management trainings for all cadres of health workers and strengthening of diagnostic capacities for the parasitological diagnosis of malaria. With respect to malaria diagnostics it should be acknowledged that despite the fact that confirmatory diagnosis is one of the key strategic case-management orientations of the Sudanese 2007-2012 National Malaria Strategy [25], by the time of this study malaria diagnostic capacities in the country relied mainly on the presence of malaria microscopy while only limited quantities of RDTs were delivered to lower level facilities without microscopy support.

In this report we present data on the availability of case-management commodities, coverage with malaria related health systems support activities and the malaria case-management practices across the Sudan 5 years after the policy change and 3 years after the launch of the national malaria strategy.

## Methods

### Survey design

The survey design was a cross-sectional, cluster sample, health facility survey undertaken in government and non-government public health facilities in 15 states of Sudan. Private health facilities were not included in the survey because they are not supplied with ACTs and malaria diagnostics by the Federal Ministry of Health. The survey was undertaken between 9^th ^and 31^st ^December 2009. The sample size was calculated at patient level assuming 50% prevalence of the primary case-management indicator, 95% confidence level, desired precision of +/- 7%, design effect of 2 and the likelihood that 50% of health facilities would not have either malaria diagnostics or ACTs in stock. Based on these parameters, an estimated sample size was 728 outpatient consultations in each age group (below and above 5 years of age) or estimating that on average 3 febrile patients can be recruited in each age group at each facility over one survey day, then the required number of surveyed facilities was 243 (728/3).

From the universe of 5,716 public health facilities in Sudan a stratified random sample taking into consideration within-country distribution of facilities by state (15 strata) and type of facilities within a state (3 strata) was drawn to ensure national representativeness. For each of 45 strata we calculated a sampling fraction within the universe of 5,716 facilities, proportional and effective sample size within the required sample of 243 facilities, and finally undertook simple random sampling for each stratum. The final effective sample size contained one additional facility because of rounding off to the proportional sample size. A cluster was defined as all outpatients' encounters between a health worker and patients occurring on a single survey day.

### Data collection

Data were collected by 72 field workers trained during 5 days until agreement of practice results between field workers and trainers was greater than 90%. In total, 36 teams were created, each composed of two field workers. The field work was supervised by 15 state coordinators who were also trainers of field workers. Three methods were used to collect data during a survey day at health facility. First, all patients presenting to the outpatient departments underwent rapid screening when they were ready to leave the facility. After obtaining written informed consent non-referred and non-pregnant patients aged 2 months and older, weighing 5 kg and above and presenting for an initial outpatient visit with fever were interviewed. Detailed information about patients' age, weight, temperature, prior use of antimalarial drugs, routine malaria diagnostics performed and results reported, medications prescribed and the key counseling and drug dispensing tasks performed during the facility visit was collected during the interviews and from the patient-held cards. Second, each health facility was assessed to determine availability of basic infrastructure, equipment, job aids, antimalarial drugs, malaria RDTs and functional malaria microscopy service. Finally, all health workers who attended to recruited patients on the survey day were interviewed at the end of the survey day to determine their demographics, pre-service and in-service training, access to treatment protocols and exposure to supervision in the preceding 6 months. Verbal informed consent was obtained from all health workers prior to the commencement of study procedures.

### Indicators and definitions

The key study indicators referred to the availability of malaria case-management commodities, health workers coverage with ACT related interventions and the malaria case-management practices which are deemed critical to the success of the AS+SP based case-management policy. The primary indicators at health facility level referred to the proportions of facilities with ACTs, other antimalarials, job aids and malaria diagnostic services on the survey day. At the health worker level the primary indicators were the proportions of health workers who received training on malaria case-management, who had access to national treatment protocols and who were exposed to supervisory visits including malaria case-management in past 6 months.

**Figure 1:**
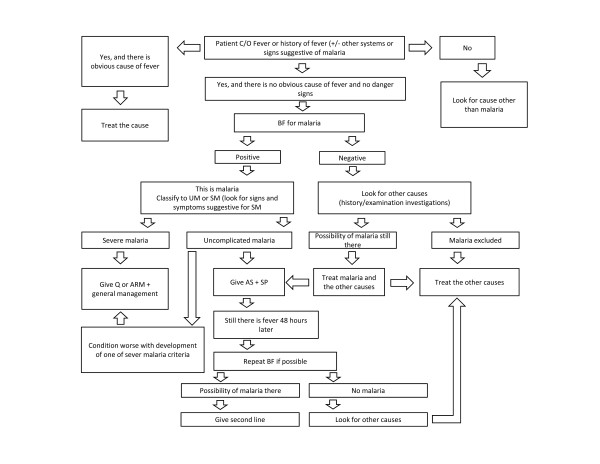
**The general plan for malaria diagnosis and treatment in Sudan**.

At the patient level primary indicators referred to febrile patients for whom malaria testing was merited and AS+SP treatment should have been considered in relation to the result of malaria test. With respect to testing indications, we included all patients with fever because national malaria guidelines are ambiguous and do not specify criteria defining other obvious causes of fever (Figure 1). Therefore our analysis included febrile, non-pregnant patients aged 2 months and older, weighing 5 kg and above and presenting for an initial outpatient visit without being referred or admitted for hospitalization. Fever was defined as axillary temperature of ≥ 37.5°C or the history of fever during the present illness. A health workers decision to refer or admit patient for hospitalization was used as proxy measure to define severity of disease and exclude patients with suspected severe disease meriting treatment with other drugs than AS+SP. The primary outcome was a composite case-management indicator and included performance of all of the following three tasks: 1) patient was tested for malaria; 2) if positive test result patient was treated with AS+SP, and 3) if negative test result patient was not treated for malaria. Despite some ambiguity of the guidelines with respect to the treatment of patients with test negative results (Figure 1), the absence of antimalarial treatment for these patients was included in the definition of the composite case-management indicator because treatment of test negative patients was considered as one of the major determinants undermining the new case-management policy. The secondary outcomes reflected individual components of the case-management including testing, treatment, dispensing and counseling practices in various patients' subgroups.

### Data management and analysis

Data were double entered by independent data entry clerks using Access 2007 (Microsoft Inc., Redmond, WA, USA). Data files were compared for errors using a verification programme and referring to original questionnaires. The analysis was performed using STATA, version 11 (StataCorp, College Station, TX, USA). Descriptive analysis was undertaken at the health facility, health worker, and patient level across all age groups and stratified for children below 5 years of age and patients 5 years and older. The presentation of the results reflected the main objective of the study, i.e. to provide national level data for which the study was sufficiently powered to obtain information with desired precision. A stepwise approach was applied in the analysis of malaria case-management practices. First, to evaluate progress of 2007-2012 national malaria strategy health workers practices are analyzed at all health facilities regardless of the availability of the case-management commodities. Second, to evaluate health workers adherence to the new policy the same analysis was restricted to the facilities where AS + SP and malaria diagnostics were available on the survey days. Finally, the quality of AS + SP dispensing and counseling practices was restricted to patients who had AS + SP prescribed and dispensed at facility. The precision of proportions (95% confidence interval [CI]) was determined adjusting for the cluster sampling at health facility level. Cluster adjusted chi-square test was used to compare proportions between patients below and above 5 years of age.

### Ethical approval

Ethical clearance for the study was obtained from the National Research Ethics Committee of the Federal Ministry of Health in Sudan (reference number 114-11-09).

## Results

### Sample description

**Figure 2:**
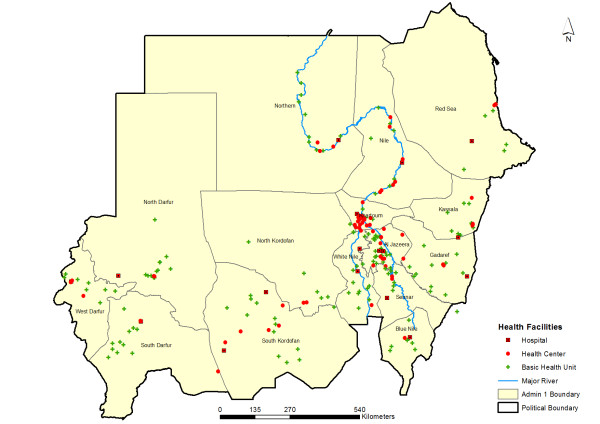
**The map of the 15 states of Sudan showing the 244 surveyed health facilities**.

The survey was undertaken at 244 health facilities (Figure 2) where 294 health workers performed 4,140 outpatient consultations on the survey days. All 244 facilities were assessed and all 294 health workers were interviewed. Of the 4,140 screened patients, the case-management practices were evaluated for 1,707 non-pregnant febrile patients weighing 5 kg and above and presenting for an initial outpatient visit. The remaining 2,433 patients (some patients may have had more than one exclusion criteria) were patients either referred (197) or admitted for hospitalization (61), pregnant women (362), follow up visits (420), aged less than 2 months (50), weighing less than 5 kg (41) and patients presenting without fever (1,365). Further 64 patients had incomplete data preventing analysis of the case-management practices. Therefore our analysis included 1,643 patients of which 425 (25.9%) were below 5 years of age and 1,218 (74.1%) were 5 years and older. At facilities with available AS+SP and malaria diagnostics on the survey day health workers' adherence was evaluated for 961 patients, of which 252 (26.2%) patients were below 5 years of age and 709 (73.8%) were 5 years and older. No health worker, adult patient or caretaker on behalf of sick child refused to participate in the study.

### Health facility and health worker readiness to implement ACT policy

Of the 244 health facilities assessed, most were basic health units (63.9%) followed by health centres (29.1%) (Table 1). The large majority (88.9%) of facilities were government owned. Nearly half (49%) had electricity, while water from any source was available at 72.1% of facilities on the survey day. A functional weighing scale was present at 58% of facilities while about half had functional thermometer (48.8%) and displayed new treatment protocol either in the format of wall or table charts (51.2%). Malaria parasitological diagnosis was provided at 125 (51.2%) facilities on the survey day, more commonly through malaria microscopy (42.2%) than non-expired RDTs (16.0%). Seventeen health facilities had both diagnostic capacities. Parasitological capacity to diagnose malaria was available in all hospitals (17/17), 79% (56/71) of health centres and 33% (52/156) of basic health units. Of interest, 12% of basic health units, 23% of health centres and 24% of hospitals had non-expired RDTs in stock. Only 13 (5%) facilities stocked expired RDTs.

**Table 1:** Characteristics of surveyed health facilities in Sudan

Health facility characteristics (N = 244)	n	%	95% CI
**Health facility type**			

Basic Health Unit	156	63.9	57.9-70.0

Health Centre	71	29.1	23.4-34.8

Hospital	17	7.0	3.8-10.2

**Health facility ownership**			

Government	217	88.9	85.0-92.9

Non-governmental organization	27	11.1	7.1-15.0

**Basic infrastructure and equipment at health facility**			

Water available	176	72.1	66.4-77.8

Electricity available	119	48.8	42.4-55.1

Functional weighing scale available	142	58.2	52.6-65.2

Functional thermometer available	119	48.8	43.6-56.4

Treatment protocol chart exposed	125	51.2	46.1-58.9

**Availability of malaria diagnostics on survey day**			

Functional microscopy service	103	42.2	35.9-48.4

Non-expired Malaria Rapid Diagnostic Tests (RDT)	39	16.0	11.3-20.6

Any functional diagnostic service (microscopy or RDTs)	125	51.2	44.9-57.5

**Availability of non-expired ACTs on the survey day**			

Any blister pack tablets of AS+SP	179	73.4	67.7-78.9

AS+SP (50 mg AS pack)	164	67.2	61.2-73.1

AS+SP (100 mg AS pack)	158	64.7	58.7-70.7

Artemether-lumefantrine (at least one pack)^a^	22	24.6	21.3-28.6

Any ACT in stock (any pack of AS+SP or AL)	181	74.2	68.7-79.7

**Availability of other antimalarials on the survey day**			

SP tablets	85	34.8	28.8-40.8

Quinine tablets	85	34.8	28.8-40.8

Quinine injections^a^	48	54.5	43.9-65.2

Artemether injections (40 or 80 mg)^a^	68	77.3	68.3-86.2

Chloroquine (any formulation)	11	4.5	1.9-7.1

^a^Analysis for these indicators restricted to 88 health centres and hospitals which are supposed to stock these items

Any non-expired blister packs of AS+SP tablets (50 or 100 mg) were in stock at 73.4% of facilities (Table 1). Conversely, expired blisters of AS+SP were found at only seven (2.9%) facilities. Non-recommended chloroquine was available at 11 (4.5%) facilities. At 88 health centres and hospitals injectable formulations of quinine and artemether were respectively available at 54.5% and 77.3% of facilities. Finally, at the same facilities at least one of the four AL weight-specific packs was in stock at only 24.6% of facilities.

Of 294 health workers who performed consultations on the survey day (Table 2), most were male (82.7%) and the most common cadres were medical assistants (43.9%), followed by doctors (26.9%) and nurses (12.6%). Doctors were the most common outpatient health workers at hospitals (67%) and health centres (52%) and the least common cadre at basic health units (6%). Over half of the health workers (52.7%) were trained on ACT use, most commonly through the NMCP in-service training programme (127/155; 81.9%). Besides the formal in-service training, brief orientation on ACT policy was provided for 42.2% of health workers. Only 23.5% of outpatient health workers were trained on RDT use. Less than half (39.7%) of health workers had access to either personal or a facility copy of national malaria treatment protocol. Finally 35.7% of outpatient health workers had received a supervisory visit in 6 months prior to the survey, and only 19.4% received a visit that included any activity on malaria case-management (Table 2).

**Table 2:** Characteristics of outpatient health workers in Sudan

Health worker characteristics (N = 294)	n	%	95% CI
**Male health worker**	243	82.7	78.3-87.0

**In-charge of health facility**	193	66.3	61.0-71.8

**Pre-service training**			

Doctor	79	26.9	21.8-32.0

Medical assistant	129	43.9	38.2-49.6

Nurse	37	12.6	8.8-16.4

Community Health Worker	37	12.6	8.8-16.4

Other cadres	12	4.1	1.8-6.4

**In-service training on malaria case-management**			

Any training based on ACT policy	155	52.7	46.9-58.4

NMCP training	127	43.2	37.5-48.9

IMCI training	71	24.1	19.2-29.1

Malaria RDT training	69	23.5	18.6-28.3

Brief ACT orientation	124	42.2	36.5-47.9

**Access to guidelines**			

Malaria treatment booklet	117	39.7	34.2-45.4

**Exposure to supervision**			

Any supervisory visit in past 6 months	105	35.7	30.2-41.2

Supervisory visit including malaria case-management	57	19.4	14.8-23.9

### Malaria diagnostic and treatment practices

Of 1,643 suspected malaria patients, 52.6% were female and the mean age of the patients was 19 years. Only 39.9% of the patients reported to the facility within two days of the febrile illness, more commonly children below 5 years (45.4%) than patients 5 years and older (38.0%). Of greater concern was that 10.1% of children and 18.4% of patients 5 years and older reported to the facility seven or more days after the beginning of the illness. Only 4.0% of patients had taken any antimalarial drug prior to the facility visit and only 19 (1.2%) patients had taken AS+SP. Axillary temperature ≥ 37.5°C was in 28.7% of patients, more commonly among children below 5 years of age (38.4%) than in patients 5 years and older (25.4%). Health workers commonly determined age of the patients (70.1%) while weight (8.9%) and temperature (3.0%) were rarely measured.

Overall, at all 244 study facilities 34.5% of febrile patients (35.3% of children vs 34.2% of patients 5 years and older; *p *= 0.723) were managed according to the composite management indicator defined as patient tested for malaria and treated with AS+SP if the test result was positive or not treated for malaria if the test result was negative. At these facilities 45.8% of febrile patients were tested without significant difference between age groups (43.8% vs 46.5%; *p *= 0.425) (Table 3). Among 752 tested patients, the test positivity rate was 40.3%, with no significant difference between children and patients 5 years and older (38.7% vs 40.8%; p = 0.647). Most patients tested (93.4%; 702/752) had microscopy performed. Among test positive patients, 62.7% of patients were treated with AS+SP, yet a substantial proportion (25.7%) received intramuscular artemether. Children below 5 years of age were more commonly treated with AS+SP (70.8% vs 60.2%; p = 0.116) while patients 5 years and older were more commonly treated with injectable artemether (28.6% vs 16.7%; *p *= 0.056). Furthermore, despite the overwhelming adherence to test negative results 15.3% of test negative patients were treated for malaria - 13.2% of children and 17.0% of patients 5 years and older (*p *= 0.336) (Table 3).

**Table 3:** Malaria diagnostic and treatment practices for febrile patients at all 244 surveyed facilities in Sudan

	Children < 5 yrs N = 425	Patients ≥ 5 yrs N = 1218	*P*-value	All age groups N = 1643	
	**n (%)**	**n (%)**		**n (%)**	**95% CI**

**Correctly managed^a^**	150 (35.3)	417 (34.2)	0.723	567 (34.5)	29.3-39.7

**Malaria test performed**	186 (43.8)	566 (46.5)	0.425	752 (45.8)	39.0-52.6

**Treatment for test positive patients**	**N = 72**	**N = 231**		**N = 303**	

**AS+SP**	51 (70.8)	139 (60.2)	0.116	190 (62.7)	55.6-69.9

**Artemether injection**	12 (16.7)	66 (28.6)	0.056	78 (25.7)	19.6-31.8

**Other antimalarials^b^**	4 (5.6)	10 (4.3)	0.754	14 (4.6)	1.1-8.2

**No AM prescribed**	5 (6.9)	16 (6.9)	0.995	21 (6.9)	3.5-10.4

**Treatment for test negative patients**	**N = 114**	**N = 335**		**N = 449**	

**AS+SP**	11 (9.6)	46 (13.7)	0.303	57 (12.7)	6.3-17.7

**Artemether injection**	3 (2.6)	8 (2.4)	0.874	11 (2.5)	1.0-3.9

**Other antimalarials^c^**	1 (0.9)	3 (0.9)	0.986	4 (0.9)	0-1.7

**Any AM prescribed**	15 (13.2)	57 (17.0)	0.336	72 (16.0)	9.3-21.3

**No AM prescribed**	99 (86.8)	278 (83.0)	0.336	377 (84.0)	77.9-90.0

**Treatment when test not done**	**N = 239**	**N = 652**		**N = 891**	

**AS+SP**	40 (16.7)	193 (29.6)	0.002	233 (26.2)	19.5-32.8

**Artemether injection**	4 (1.7)	32 (4.9)	0.049	36 (4.0)	1.7-6.3

**Other antimalarials^d^**	6 (2.5)	11 (1.7)	0.251	17 (1.9)	0.6-3.2

**No AM prescribed**	189 (79.1)	416 (63.8)	0.001	605 (67.9)	60.9-74.9

^a^Defined as performance of all of the following three tasks: 1) patient tested for malaria; 2) if positive test result treated with AS+SP, and 3) if negative test result not treated for malaria.

^b^Other antimalarial treatments include: SP (5), QN (5) and AL (4)

^c^Other antimalarial treatments include: SP (3), QN (1)

^d^Other antimalarial treatments include: SP (5), QN (7) and CQ (5)

Compared to the practices observed at all 244 health facilities, some of the indicators significantly improved at 107 (43.9%) facilities where malaria diagnostic services (microscopy or RDTs) and AS+SP were available on the survey day. The composite case-management performance increased from 34.5% to 50.5% while testing rates for febrile patients increased from 45.8% to as high as 66.8% of patients tested. Interestingly, at these facilities where AS+SP was in stock, the pattern of treatment practices for test positive and test negative patients showed only minor changes compared to observations at all health facilities (Tables 3 and 4).

**Table 4:** Malaria diagnostic and treatment practices for febrile patients at 117 facilities with available AS + SP and malaria diagnostic services on survey days in Sudan

	Children < 5 yrs N = 252	Patients ≥ 5 yrs N = 709	*P*-value	All age groups N = 961	
		
	n (%)	n (%)		n (%)	95% CI
**Correctly managed^a^**	120 (47.6)	365 (51.5)	0.325	485 (50.5)	44.5-56.4

**Malaria test performed**	154 (61.1)	488 (68.8)	0.045	642 (66.8)	59.6-74.0

**Treatment for test positive patients**	**N = 59**	**N = 190**		**N = 249**	

**AS+SP**	40 (67.8)	120 (63.2)	0.519	160 (64.3)	56.3-72.2

**Artemether injection**	10 (17.0)	50 (26.3)	0.174	60 (24.1)	17.6-30.5

**Other antimalarials^b^**	4 (6.8)	5 (2.6)	0.278	9 (3.6)	0.4-6.8

**No AM prescribed**	5 (8.5)	15 (7.9)	0.863	20 (8.0)	3.8-12.2

**Treatment for test negative patients**	**N = 95**	**N = 298**		**N = 393**	

**AS+SP**	11 (11.6)	42 (14.1)	0.574	53 (13.5)	7.1-19.8

**Artemether injection**	3 (3.2)	8 (2.7)	0.791	11 (2.8)	1.1-4.5

**Other antimalarials^c^**	1 (1.1)	3 (1.0)	0.969	4 (1.0)	0-2.0

**Any AM prescribed**	15 (15.8)	53 (17.8)	0.657	68 (17.3)	10.5-24.1

**No AM prescribed**	80 (84.2)	245 (82.2)	0.657	325 (82.7)	75.9-89.5

**Treatment when test not done**	**N = 98**	**N = 221**		**N = 319**	

**AS+SP**	8 (8.2)	31 (14.0)	0.278	39 (12.2)	4.4-20.0

**Artemether injection**	1 (1.0)	5 (2.3)	0.389	6 (1.9)	0-3.7

**Other antimalarials^d^**	1 (1.0)	0 (0)	0.111	1 (0.3)	0-0.9

**No AM prescribed**	88 (89.8)	185 (83.7)	0.320	273 (85.6)	76.7-94.4

^a^Defined as performance of all of the following three tasks: 1) patient tested for malaria; 2) if positive test result treated with AS+SP, and 3) if negative test result not treated for malaria

^b^Other antimalarial treatments include: SP (3), QN (2) and AL (4)

^c^Other antimalarial treatments include: SP (3), QN (1)

^d^Other antimalarial treatments include: SP (1)

### Quality of ACT dispensing and counseling

The performance of five dispensing and counseling tasks was evaluated for 347 patients who had AS+SP prescribed and dispensed at health facilities (Table 5). The majority of the patients were explained how to take AS+SP drugs at home (86.5%) and advised to complete all AS+SP doses (60.7%), however only 2.9% of patients had the first dose administered at the health facility, 6.1% were weighed and the advice on what patients should do in case of vomiting was rarely provided (6.4%). Children below 5 years of age were more commonly weighed than patients 5 years and older (13.9% vs 4.0%; *p *= 0.006) and advice on vomiting was more commonly provided for children than patients 5 years and older (12.5% vs 4.8%; *p *= 0.009) (Table 5).

**Table 5:** Quality of AS+SP dispensing and counseling in Sudan

	Children < 5 yrs N = 72	Patients ≥ 5 yrs N = 275	*P*-value	All age groups N = 347	
		
	n (%)	n (%)		n (%)	95% CI
**Explained dosing schedule**	64 (88.9)	236 (85.8)	0.657	300 (86.5)	82.8-90.1

**Advised to complete all doses**	52 (73.2)^a^	158 (57.5)	0.070	210 (60.5)^a^	55.5-65.9

**Patient weighed**	10 (13.9)	11 (4.0)	0.006	21 (6.1)	3.5-8.6

**First dose administered at HF**	0 (0)	10 (3.6)	0.115	10 (2.9)	1.1-4.6

**Explained what to do if vomiting**	9 (12.5)	13 (4.8)^b^	0.009	22 (6.3)^b^	3.8-8.9

^a^Denominator does not include one patient with missing value

^b^Denominator does not include two patients with missing values

## Discussion

Our facility-based evaluation of the malaria case-management under the ACT policy in the 15 states of Sudan provides a number of observations relevant for future implementation activities under the 2007-2012 National Malaria Strategy.

### Health systems support to implement ACT based malaria case-management

The critical pre-requisite for effective implementation of any treatment policy is universal availability of recommended, non-expired medicines as well as discontinued provision of obsolete therapies. The findings of this study revealed that chloroquine monotherapy had largely been successfully phased out from Sudanese facilities (absent at 95% of facilities), the presence of expired ACTs was nearly non-existent (3%) while recommended first-line therapy (AS+SP) was in stock at 73% of facilities and second-line therapy (AL) at only 25% of facilities. The finding that over one-quarter (26%) of facilities stocked no ACTs on the survey days is worrisome especially in comparison with prior studies in Sudan reporting nearly universal availability of recommended first-line antimalarials under the chloroquine policy [26,27]. However, the magnitude of ACT stock-outs found in this study is not unique to Sudan - nearly equal results were recently reported from Kenya where 25% of public facilities had ACT stock-outs [3] and similar findings were reported from Uganda [18]. Future studies evaluating qualitative and quantitative characteristics of ACT supply chain are required in Sudan to better understand causes of ACT stock outs.

The second pre-requisite important for effective implementation of malaria case-management includes the capacity at health facilities to undertake malaria testing. Our survey revealed that currently about half (51%) of the public facilities in Sudan provide malaria diagnostic services, largely based on microscopy. While the relatively low availability of malaria diagnostics is understandable at basic health units (33%) where only limited diagnostic capacities have been deployed in the past it should be however noted that parasitological diagnosis was absent at 21% of health centres - the level of care traditionally providing laboratory support in Sudan. To rapidly increase the coverage of health facilities with malaria diagnostic services a focus on large scale procurement and supply of RDTs accompanied with the quality assurance activities to those facilities where malaria microscopy service is currently not available will fill an important gap. Simultaneously, at facilities with existent microcopy the strengthening of the quality of this service is the utmost priority. The quality assurance activities supporting both, RDTs and malaria microscopy, will be critical determinants to ensure routine accuracy of RDTs and overcome deficiencies in the quality of malaria microscopic services that have been previously reported in Sudan [28,29] as well as in the region [30,31]

Finally the provision of ACTs and diagnostics requires a package of health systems support activities for health workers which are necessary to implement, reinforce and maintain effective malaria case-management practices. Our findings revealed gaps in the coverage of health workers with malaria related health systems support activities; 47% of outpatient health workers had not attended ACT based case-management training, 76% had not been trained in the use of RDTs, 60% had no access to treatment protocols, 64% had not received any supervisory visit and importantly only 19% received a visit that included any activity related to malaria case-management. An opportunity to rapidly increase health workers exposure to these activities lies in the large scale RDT implementation which should be accompanied with broader and comprehensive malaria case-management in-service training, dissemination of job aids, post-training follow up and structured supervisory visits and performance monitoring.

### Malaria case-management practices

We have defined correct management of febrile patients from malaria perspective considering performance of the three minimum criteria determining success of ACT based policy based on confirmatory diagnosis [20]. These included 1) malaria testing, 2) treatment of test positive results with recommended AS+SP and 3) withdrawal of antimalarial treatment for test negative patients. Our findings revealed that at all study facilities 35% of febrile patients are currently managed according to the composite indicator, with little difference between age groups. At the same facilities 46% of febrile patients are tested. While analysis based on all study facilities is useful to evaluate overall progress of the case-management practices under the 2007-2012 National Malaria Strategy, it does not consider the absence of ACTs and diagnostics at health facilities - the main determinant precluding case-management practices. Therefore, to evaluate health workers adherence to the new policies practices at 117 (48%) facilities where both, malaria diagnostic services and AS+SP, were available on the survey day were evaluated separately. At these facilities health workers performed all three tasks defining our composite indicator for a significantly higher proportion (51%) of febrile patients compared to analysis based on all facilities.

There are three levels of discordance contributing to the non-adherent case-management practices at facilities with available commodities. First, not all febrile patients are tested for malaria, however, 67% of patients parasitologically diagnosed is significantly higher compared to 43% reported in similar studies in Kenya [32], 40% in Uganda [12], 31% in Angola [9] 27% in Zambia [5] and 27% in Tanzania [7]. The possible explanation of higher testing rates at facilities with malaria diagnostics in Sudan compared to other countries could be due to the presence of more qualified outpatient staff such as medical doctors and long term establishment of malaria microscopy in the country traditionally promoting, wherever possible, malaria testing across all age groups and malaria endemicities. The future priority for policy implementers in Sudan should be improvement in testing rates across all facilities.

The second level of discordance refers to patients with positive test result where only 64% of patients at facilities with AS+SP in stock, are treated with recommended first line therapy for uncomplicated malaria. At these facilities an important observation was the relatively wide-spread use of injectable artemether monotherapy, a practice not promoted globally on the grounds that it might promote the development of resistance to artemisinine derivatives [33]. In this category of patients, we also observed tendency towards higher use of oral AS+SP in children while use of injectable artemether was more common for older children and adults, the finding reflecting previous report from Khartoum where irrational injectable therapy under the chloroquine policy was common and indeed prevailing in adults [26]. Injectable antimalarials have a long history in Sudan [26,34] and may be due to a combination of factors including the lack of appropriate training of health workers, lack of public education and patients' demands and beliefs. Future educational and supervisory interventions targeting health workers, as well as those targeting the broader public, should urgently address the problem of inappropriate use of injectable artemether.

The third and the lowest level of discordance was the observation made amongst those patients with a negative test result who were still treated with an antimalarial (17%). While this remains imperfect, this level of inappropriate prescription is substantially lower than reported under larger scale evaluations in other national settings [5,7,10,11,13,35]. Interestingly, this relatively low level of disregard of negative test results existed despite the ambiguity of national guidelines allowing possibility of antimalarial treatment for test negative patients (Figure 1). International recommendations have recently changed to recommend treatment of only test positive patients and Sudanese guidelines should be also amended along these lines to facilitate elimination of these irrational practices (WHO 2010).

Finally, adequate ACT dispensing and counseling practices deserve attention as these tasks underpin good clinical practice necessary to ensure high rates of patients' adherence and treatment success [36-38]. In our study we found that the majority of patients were explained the dosing schedule (87%) and advised to complete the dose (61%), however, the practices of administering the first dose of AS+SP at the facility, weighing patients and providing advice to patients what to do in case of vomiting was rarely performed (3-6%). Suboptimal dispensing and counseling practices found in our study concur with the findings reported previously in Sudan [27,39], as well as from several studies across Africa [15,19]. The reasons for these suboptimal practices are not clear and demand further qualitative research including the potential effects of lack of potable water on administering drugs at facilities and the effects of AS+SP blister packages on the provision of replacement dose in case of vomiting.

### Study limitations

Several study limitations should be mentioned. First, the presence of study teams may have introduced the Hawthorn effect resulting in better performance of health workers on survey days than usual [40,41]. To minimize this possibility we restrained from more "invasive" study procedures such as observation of consultations and limit patient level data collection only to patient's exit interviews. Second, the evaluation was undertaken from malaria case-management perspective and did not include data collection methods such as clinical re-examinations and direct observations to evaluate clinical assessment practices for non-malaria febrile patients which would provide an additional light to some of the practices. Finally, the appropriate drug dosage component of antimalarial treatments was not analyzed due to high rates of incomplete dosage prescriptions.

## Conclusions

Five years following the shift of the Sudanese treatment policy to ACTs and three years before the end of the 2007-2012 Malaria Strategic Plan the chloroquine monotherapy was successfully phased out from public health facilities, however, the availability of recommended first-line (AS+SP) and second-line (AL) therapies was suboptimal. Malaria diagnostic capacities were present at half of the facilities largely dependent on the availability of malaria microscopy. There is an important gap in the health workers and health facility coverage with the in-service training, job aids and in particular with malaria related supervisory activities. With respect to case-management practices less than half of febrile patients are currently tested, however at facilities with available diagnostics the testing rates were reasonably high with two-thirds of the patients tested for malaria. The prevailing treatment practice for test positive patients is in line with national protocols recommending AS+SP, however worryingly one-quarter of the patients is treated with injectable artemether monotherapy. The relatively low prescription rates of antimalarial treatments for test negative patients compared to other countries are encouraging. Yet ACT dispensing and counseling practices were largely suboptimal. Overall, at all health facilities 35% of febrile patients were tested and treated according to test result while at facilities with available commodities 51% of febrile patients met the same criteria. Future qualitative research is required to better understand deficiencies of the supply chain and clinical practices and the forthcoming large scale implementation of RDTs, using the findings of this survey and pending qualitative research, should present an opportunity not only to expand coverage of testing capacities to the facilities where malaria diagnostics are currently not available, but also to implement effective support package of the interventions to bridge the health systems gaps and do further corrective measures to mainstream the recommended case-management. However, the adequate availability of ACTs and diagnostics at the point of care will ultimately determine the success of case-management policies in Sudan.

## Competing interests

DZ and RWS have received honoraria from Novartis Pharma for presenting and chairing, respectively, at their national malaria control programme best practice workshops in Africa.

## Authors' contributions

AI and KAE were involved in design and development of survey tools, implementation of survey, and collection of data and drafting of the manuscript. AMN designed the study, collected and cleaned the data and participated in analysis and drafting the manuscript. SG reanalyzed the data and participated in drafting the manuscript. DZ participated in study design and development of survey tools, analysis and drafting of the manuscript. RWS participated in the conception of the study questions, provided overall scientific direction and was involved in the analysis and drafting of the manuscript. All authors have read and approved the final manuscript.

## Pre-publication history

The pre-publication history for this paper can be accessed here:

http://www.biomedcentral.com/1471-2458/12/11/prepub
